# Impact of Luteinized Unruptured Follicles on Clinical Outcomes of Natural Cycles for Frozen/Thawed Blastocyst Transfer

**DOI:** 10.3389/fendo.2021.738005

**Published:** 2021-10-21

**Authors:** Song Li, Lokwan Liu, Tian Meng, Benyu Miao, Mingna Sun, Canquan Zhou, Yanwen Xu

**Affiliations:** ^1^ Reproductive Medicine Center, First Affiliated Hospital, Sun Yat-sen University, Guangzhou, China; ^2^ Guangdong Provincial Key Laboratory of Reproductive Medicine, First Affiliated Hospital, Sun Yat-sen University, Guangzhou, China

**Keywords:** luteinized unruptured follicle, ovulation, frozen/thawed blastocysts transfer, clinical outcomes, LH surge

## Abstract

**Objective:**

To investigate the impact of luteinized unruptured follicles (LUF) on clinical outcomes of frozen/thawed embryo transfer (FET) of blastocysts.

**Methods:**

In this retrospective cohort study, 2,192 patients who had undergone blastocyst FET treatment with natural cycles from October 2014 to September 2017 were included. Using propensity score matching, 177 patients diagnosed with LUF (LUF group) were matched with 354 ovulating patients (ovulation group). The LUF group was further stratified by the average LH peak level of 30 IU/L. Clinical pregnancy rate and live birth rate were retrospectively analyzed between the LUF and ovulation groups, as well as between LUF subgroups.

**Results:**

After propensity score matching, general characteristics were similar in the LUF and ovulation groups. Clinical pregnancy rate in the LUF group was significantly lower than that in the ovulation group (47.46 *vs.* 58.76%, respectively, adjusted *P *= 0.01, OR 0.60, 95% CI 0.42–0.87). However, no significant difference was detected in live birth rate, although it was lower in the LUF group (43.50 *vs.* 50.00%, adjusted *P *= 0.19, OR 0.76, 95% CI 0.51–1.14). In the LUF subgroup analysis, both clinical pregnancy rate (43.02 *vs.* 62.30%, adjusted *P *= 0.02, OR 0.45, 95% CI 0.23–0.87) and live birth rate (37.21 *vs.* 59.02%, adjusted *P *= 0.01, OR 0.40, 95% CI 0.20–0.78) in the LH <30 IU/L subgroup were significantly lower than those in the LH ≥30 IU/L subgroup.

**Conclusion:**

LUF negatively affected clinical outcomes of frozen/thawed embryo transfer of blastocysts, particularly when the LH surge was inadequate.

## Introduction

With the increasing application of assisted reproductive technology (ART) worldwide, the advantages and disadvantages of various methods related to ART have been widely discussed (1). In frozen/thawed embryo transfer (FET) cycles, either a hormone replacement therapy cycle (HRT) or natural ovulation cycle (NC) is used for endometrial preparation. Although a number of studies demonstrate that FET significantly improves clinical outcomes and allows consecutive embryo transfers (2, 3), it is associated with a higher risk of pregnancy-related hypertensive disorders, post-term delivery, macrosomia, and other adverse obstetrical or prenatal outcomes, especially for HRT cycles (4). The difference between HRT and NC is the absence of a corpus luteum, which produces crucial hormones for implantation, placentation, and pregnancy maintenance (5). Recent studies demonstrate that the absence of a corpus luteum has a negative impact on vascular health, leading to insufficient cardiovascular adaptation in early pregnancy, and contributing to an increased risk of preeclampsia (6, 7). From this point of view, endometrial preparation in natural ovulation cycles with a corpus luteum present appear to be safer. However, inconvenience exists in NC-FET. The development of a dominant follicle and luteinizing hormone (LH) levels need to be frequently detected. If the dominant follicle fails to rupture after the LH surge or human chorionic gonadotropin (hCG) trigger in a natural cycle, this is named a luteinized unruptured follicle (LUF). The impact of LUF on FET clinical outcome is still uncertain.

LUF syndrome (LUFS) is characterized by a normal menstrual period and biphasic basal body temperature but without ovulation after the LH peak. It results from unknown reasons and is considered to be a cause of infertility in women. LUFS was first described by Jewelewicz (8), and then first diagnosed using laparoscopy by the absence of an ovulation stigma and the demonstration of lower concentrations of estrogen and progesterone in peritoneal fluid compared with normal ovulatory cycles in 1978 (9). Currently, this diagnosis is usually made based on ultrasound examination combined with a serum LH test. The incidence of LUF is estimated to be 5–10% in women of childbearing age but 25% in infertile women (10, 11). Deficient luteal function in LUF cycles has been observed with significantly lower mid-luteal progesterone levels and shorter luteal phase duration (12). It is well acknowledged that progesterone is essential for endometrial decidualization, which plays an important role in embryo implantation (13). Furthermore, it has been shown that luteal phase deficiency may lead to infertility or early pregnancy loss (14). As a result, LUF might negatively affect embryo implantation or ongoing pregnancy in NC-FET. However, evidence of the impact of LUF on clinical outcomes of FET is lacking. In addition, it remains elusive whether a stronger luteal phase support could compensate the deficient luteal function in LUF. To the best of our knowledge, there is only one study, by Wang et al. (15), that has investigated the impact of LUF on pregnancy outcomes of frozen/thawed cleavage embryo transfer, and it found LUF did not affect the clinical outcomes of FET. It should be pointed out that a slow freezing and rapid thawing method was used in the study; therefore, the conclusion could not be applied to the more popular use of vitrification for embryo cryopreservation. Moreover, with a tendency toward blastocyst culture and elective single embryo transfer, data from blastocysts might be more valuable.

In this study, we retrospectively analyzed the clinical outcomes of frozen/thawed blastocyst transfer between LUF and ovulation cycles. The purpose of this study was to investigate whether there is a negative impact of LUF on clinical pregnancy rate and live birth rate.

## Material and Methods

**Figure 1:**
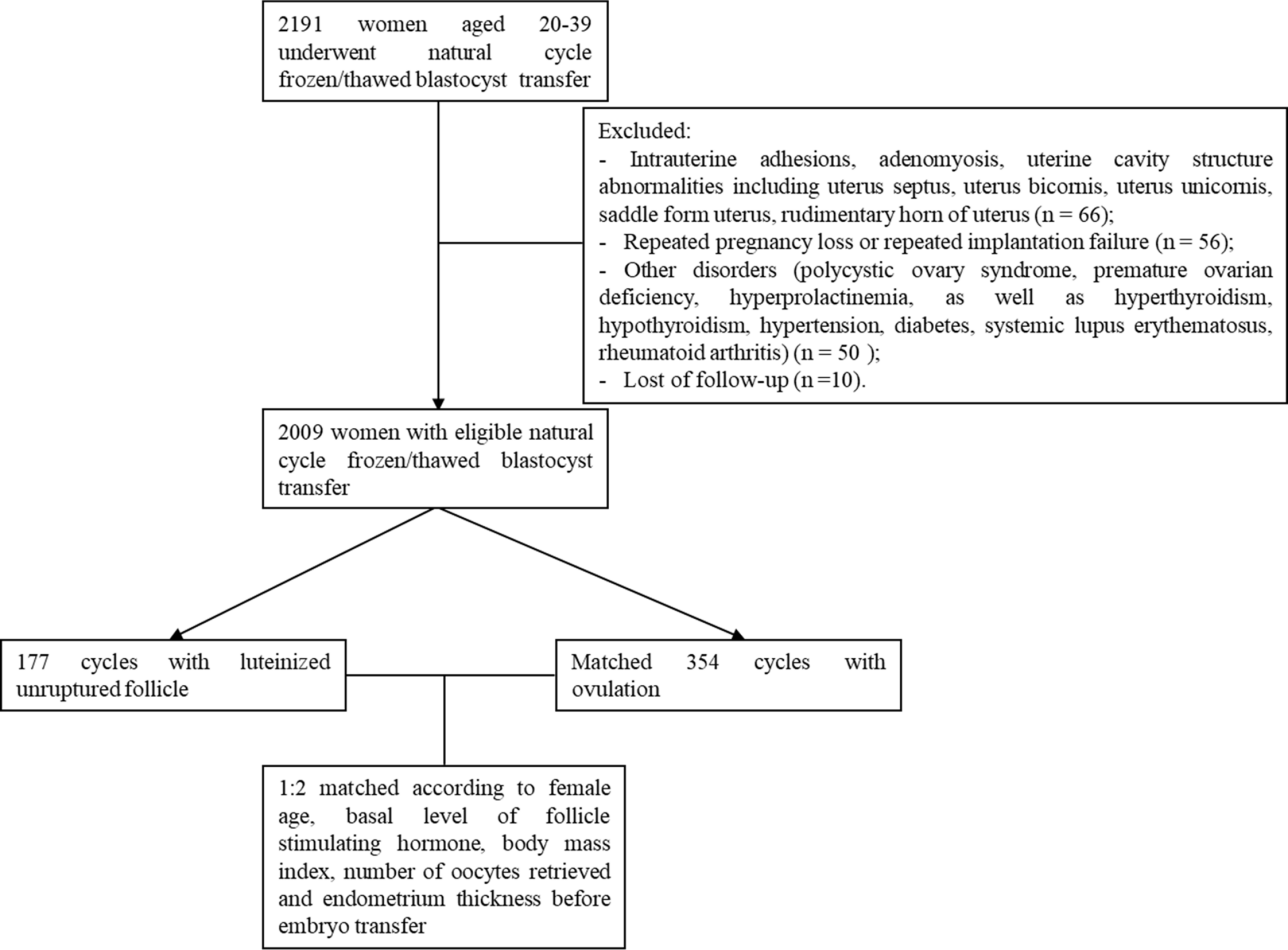
Flowchart of the cohort.

Patients with regular menstrual cycles are usually recommended NCs during FET cycles in our clinic. Here, we included 2,192 patients aged 20–39 y who had undergone blastocyst FET treatment with natural cycles at the Reproductive Centre of the First Affiliated Hospital, Sun Yat-sen University, Guangzhou, China, between October 1, 2014 and September 30, 2017. Only one cycle was involved from each patient. If one case had several cycles, only the first NC-FET cycle was included. Patients who underwent pre-implantation genetic testing (PGT) were also included. Patients who had been diagnosed with repeated pregnancy loss (n = 56) or intrauterine adhesions (n = 29), adenomyosis (n = 6), uterine cavity structure abnormalities (n = 31) such as uterine mediastinum, and other disorders [polycystic ovary syndrome (n = 15), premature ovarian deficiency (n = 3), hyperprolactinemia (n = 20), hyperthyroidism (n = 11), hypothyroidism (n = 2), hypertension (n = 5), diabetes (n = 1), systemic lupus erythematosus (n = 2), rheumatoid arthritis (n = 1)] were excluded. Ten patients were lost to follow-up (n = 10). A total of 2,009 infertile women underwent NC-FET and met the above criteria. Of them, 177 (8.81%) cycles with LUF composed the LUF group. These cycles were matched with 354 ovulation cycles (ovulation group) according to maternal age, basal level of follicle stimulating hormone (FSH), body mass index (BMI), number of oocytes retrieved in controlled ovarian stimulation (COS) cycles, and endometrium thickness before embryo transfer by using propensity score matching (1:2 matching with the 0.1 caliper value). Finally, 531 cycles were included. The LH peak value was defined as four-fold higher than the basal LH level. Stratified by the average LH peak level of 30 IU/L, LUF cycles were further divided into subgroups. Among 177 LUF cycles, 86 cycles were in the LH <30 IU/L subgroup, and 61 cycles in the LH ≥30 IU/L subgroup. The LH surge of the remaining 30 cycles was not detected. The inclusion and exclusion details of the analyzed cohort are shown in **Figure 1**.

This study was approved by the Research Ethics Committee of the First Affiliated Hospital, Sun Yat-sen University. The ovarian stimulation protocols of fresh cycles from which blastocysts developed are described in our previous study (16). Briefly, gonadotrophin dose was chosen based on individual patient parameters and adjusted according to ovarian response. When at least two follicles had reached 18 mm, 5,000–10,000 IU hCG was administered to induce final oocyte maturation. Transvaginal oocyte retrieval was then scheduled 36 h later. Fertilization was performed by conventional *in vitro* fertilization (IVF) or by intracytoplasmic sperm injection (ICSI), and all embryos were cryopreserved using vitrification. All relevant procedures in the embryo laboratory and PGT lab are described in our previous studies (16, 17).

FET was performed in a natural cycle for all included patients. Briefly, transvaginal ultrasound was performed from Day 10 of the menstrual cycle to ensure a follicle had been selected. When the diameter of the dominant follicle reached 14 mm, patients were asked to monitor their urine LH surge daily. Serum levels of LH, estrogen, and progestogen were detected when the urine LH test was positive or when the diameter of the dominant follicle reached 18 mm. In addition, transvaginal ultrasound was performed daily until ovulation or confirmation of LUF. Few patients (10 in the ovulation group and 24 in the LUF group) were prescribed hCG to induce ovulation according to the clinicians’ preference. Final ovulation induction for these patients was achieved by administration of 5,000 or 10,000 IU hCG when endometrial thickness reached 8 mm or more and an 18 mm follicle was present on ultrasound. Serum hormone levels were also tested.

The diagnostic criteria of LUF were as follows (18, 19): (1) a normal follicle continued to grow in size after the LH surge with a thickened follicular wall and increased internal resonance; in addition, extensive intrafollicular bright spots might be detected after 2 to 4 days; (2) rapid enlargement to a size of 30–35 mm in diameter with strong internal resonance, persistent till the next cycle or even longer intrafollicular echoes which might be (i) low, medium, or high; (ii) echoless; (iii) diffuse and linear; (iv) reticular or band-like. For confirmation of LUF, at least two consecutive transvaginal ultrasound scans by two investigators on different day after LH surge were required.

Blastocyst transfer was scheduled on the 5^th^ day of ovulation. For the LUF group, the day with a progestogen level >1.0 ng/ml after LH surge was assumed to be the ovulation day. In the ovulation group, the day of ovulation was indicated by disappearance of the dominant follicle and appearance of the corpus luteum with transvaginal ultrasound performed by experienced clinicians. According to the Gardner grading system, a good quality embryo transfer was considered to have taken place when a blastocyst had an expansion grade of ≥3 (expanded blastocyst to hatched blastocyst), an inner cell mass grade of A or B, and a trophectoderm grade of A or B, i.e., 3BB or superior. According to the clinician’s preference, one out of four luteal phase support (LPS) protocols could be given after confirmation of ovulation or LUF in our clinic: (1) only progesterone [oral dydrogesterone (20 mg daily) and/or vaginal progesterone, such as Utrogestan (600 mg daily) or 8% Crinone (90 mg daily), and/or intramuscular (i.m.) injected progesterone (40–60 mg daily)]; (2) i.m. injected hCG (2,000 IU every 3 days) combined with daily progesterone and/or oral estradiol valerate (3 mg/d); (3) progesterone combined with oral estradiol valerate; (4) only i.m. hCG. LPS protocols prescribed in the fresh embryo transfer cycles, such as vaginal progesterone with 600 mg Utrogestan daily; 90 mg 8% Crinone per day; 40–60 mg i.m. progesterone daily; or injected hCG, were defined as stronger LPS protocols, as it was mandatory to supplement luteal function in the fresh embryo transfer cycles. LPS would persist until 10–11 weeks of gestation if pregnancy was confirmed.

Serum hCG levels were determined 12–14 days after embryo transfer. Transvaginal ultrasonography was performed at 7 weeks of gestation. All pregnancies were followed up to 3 months after delivery or pregnancy loss.

### Statistical Analysis

The primary outcomes in this study were clinical pregnancy rate and live birth rate, and secondary outcomes were implantation rate and early pregnancy loss rate. Clinical pregnancy rate was defined as the ratio of cycles with a confirmed fetal heart and gestational sac at 7 weeks of gestation in all FET cycles. Implantation rate was calculated as the total number of gestational sacs divided by the total number of embryos transferred. Early pregnancy loss rate was the number of pregnancies lost before 12 weeks of gestation divided by the number of clinical pregnancies. Live birth rate was defined as the ratio of cycles with live births of all FET cycles. Since the difference of clinical outcomes between LUF and ovulation patients was largely unknown, we included all FET cycles with LUF and matched them with twofold ovulation cycles.

Data are presented as mean ± SD or median (range) for continuous variables and as n (%) for categoric variables. The differences in continuous variables between the case and control groups were analyzed by means of independent-sample *t-*test if data followed normal distributions and the variances between the two groups were equal; otherwise, the Mann–Whitney U-test was applied. The chi-square test for categoric variables was used for each group. Binary logistic regression was used to control luteal support protocols (as categorical variables including seven categories: dydrogesterone, injected progesterone, vaginal progesterone, vaginal progesterone, mixed progesterone, progestogens + estrogen, hCG + estrogen/progestogens, and only hCG) and the number of embryos transferred (as numerical variables). While performing subgroup analysis, the number of matured oocytes retrieved in COS cycles was adjusted by binary logistic regression. SPSS version 25.0 was used for data analysis. A *P*-value of <0.05 indicated statistical significance.

## Results

Baseline characteristics are listed in **Table 1**. There were no significant differences with regards to the mean maternal age, body mass index (BMI), course or type of infertility, basal FSH or PRL, as well as gravidity and parity history between the LUF and ovulation groups. There were no significant differences in terms of numbers of total oocytes and embryos, as well as fertilization protocols of fresh oocyte retrieval cycles. The quality of transferred embryos was compatible between the two groups.

**Table 1:** Baseline characteristics between the LUF group and ovulation group.

	LUF	Ovulation	*P*-value
Cycles	177	354	
Female age (y)	31.42 ± 3.59	31.78 ± 3.74	0.30
BMI (kg/m^2^)	21.29 ± 2.65	20.91 ± 2.45	0.10
Course of infertility (y)	4.09 ± 2.90	4.05 ± 2.80	0.86
Primary infertility, n (%)	95 (53.67)	186 (52.54)	0.81
Parity ≥1, n (%)	49 (27.68)	123 (34.75)	0.10
Cause of infertility^a^, n (%)			0.12
Fallopian tube or pelvic factors	85 (49.42)	143 (41.81)	
Male factors	35 (20.35)	103 (30.12)	
Mix factors (both male and female)	42 (24.42)	79 (23.10)	
Unexplained infertility	10 (5.81)	17 (4.97)	
Basal FSH (IU/L)	5.61 ± 1.48	5.60 ± 1.38	0.94
Basal PRL (ng/ml)	16.85 ± 8.44	17.46 ± 9.77	0.48
Times of pregnancy loss, n (%)			0.78
0	154 (87.01)	314 (88.70)	
1	18 (10.17)	33 (9.32)	
2	5 (2.82)	7 (2.98)	
ICSI fertilization, n (%)^b^	71 (40.11)	155 (43.79)	0.42
COS cycle			
Number of oocytes retrieved	16.54 ± 7.22	17.30 ± 7.63	0.27
Number of mature oocytes	14.71 ± 6.19	15.06 ± 6.52	0.55
Number of available embryos	6.51 ± 3.03	6.94 ± 3.27	0.14
Number of good embryos	5.33 ± 3.29	5.52 ± 3.51	0.54
Number of frozen embryos	5.38 ± 3.40	5.81 ± 3.73	0.19
PGT cycles (%)	23 (12.99)	50 (14.12)	0.72
PGT-A cycles, n (%)	8 (4.52)	20 (5.65)	0.58
Number of FET attempts, n (%)			0.12
1	130 (73.45)	274 (77.40)	
2	37 (20.90)	51 (14.41)	
>2	10 (5.65)	29 (8.19)	
Endometrium thickness (mm)	10.46 ± 2.07	10.53 ± 2.01	0.69
Number of embryos transferred	1.51 ± 0.50	1.43 ± 0.50	0.09^*^
Period of embryos thawed (months)	5 (1, 80)	4 (1, 83)	0.20^*^
Embryo quality			0.65
Good, n (%)	125 (70.62)	262 (74.01)	
Moderate, n (%)	25 (14.13)	41 (11.58)	
Poor, n (%)	27 (15.25)	51 (14.41)	

a Five patients in the LUF group and 12 patients in the ovulation group underwent PGT due to genetic factors and were fertile.

b Two patients in the ovulation group had two blastocysts transferred from a half-ICSI protocol. ^*^Mann–Whitney U test.

Regarding the LPS protocols (**Table 2**), progesterone or hCG+ estrogen/progestogens were most frequently used in both groups. However, a higher percentage of cycles with i.m. or vaginal progesterone [90 mg Crinone (8%) daily or 200 mg Utrogestan three times daily] were prescribed in the LUF group, while more cycles with only oral dydrogesterone (20 mg daily) were used in the ovulation group. In addition, more cycles in the LUF group used both progesterone and estrogen treatments.

**Table 2:** Luteal support protocols of LUF group and ovulation group.

	LUF	Ovulation	*P*-value
Cycles	177	354	
Luteal support protocol^a^			<0.001
Progestogens, n (%)	78 (44.07)	191 (54.26)	<0.001^b^
Dydrogesterone, n (%)	29 (16.38)	152 (43.18)	
Injected progesterone, n (%)	23 (13.00)	21 (5.97)	
Vaginal progesterone, n (%)	12 (6.78)	5 (1.42)	
Mixed, n (%)	14 (7.91)	13 (3.69)	
Progestogens + estrogen, n (%)	14 (7.91)	5 (1.42)	
hCG + estrogen/progestogens, n (%)	83 (46.89)	134 (38.07)	
Only hCG, n (%)	2 (1.13)	22 (6.25)	

a Three patients in the ovulation group received no luteal support.

b P-value for comparison of different types of progestogens used between two groups.

We then examined serum levels of sex hormones on the day of LH surge and the day of embryo transfer (**Table 3**). Results showed that the serum level of the LH peak, progesterone levels on the day of LH surge, and the day of embryo transfer in the LUF group were all significantly lower than those in the ovulation group. In addition, the mean diameter of dominant follicles on the day of LH surge was found to be smaller in the LUF group (*P* < 0.001). However, level of estrogen on embryo transferred day was significantly higher in the LUF group (*P* = 0.002).

**Table 3:** Serum levels of sex hormones on LH surge day and embryo transfer day.

	LUF	Ovulation	*P*-value
Cycles	177	354	
LH level on LH surge day (IU/L)	30.52 ± 15.37	38.11 ± 16.33	<0.001
E_2_ level on LH surge day (pg/ml)	242.31 ± 91.84	258.53 ± 92.92	0.09
Progesterone level on LH surge day (ng/ml)	0.67 ± 0.25	0.77 ± 0.24	<0.001
Diameter of dominant follicle (mm)	17.47 ± 2.15	18.18 ± 1.89	<0.001
E_2_ level on transfer day (pg/ml)	161.54 ± 73.21	143.39 ± 56.49	0.002
Progesterone level on transfer day (ng/ml)	12.54 ± 6.40	13.87 ± 5.21	0.01
Median (Range) (ng/ml)	11.40 (2.30, 61.90)	13.35 (3.50, 42.90)	

**Figure 2:**
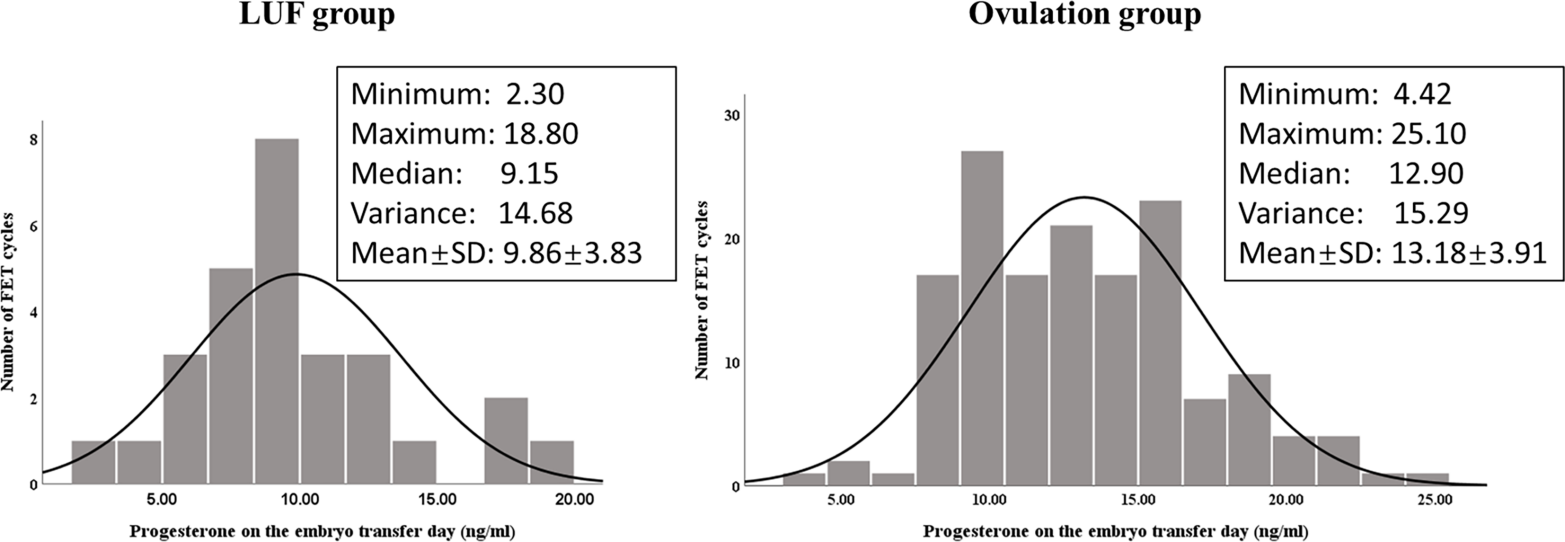
Progesterone level (ng/ml) distribution in cycles treated with only oral dydrogesterone (20 mg daily) for LPS in the LUF group (n = 29) and the ovulation group (n = 152). The averages of progesterone were significantly different between the two groups (*P* < 0.001).

For avoiding the potential effect of various LPS protocols, we further compared the progesterone level on the embryo transfer day in cycles treated with only oral dydrogesterone (20 mg daily) for LPS, as oral dydrogesterone does not affect the progesterone level in serum. We found that the difference of progesterone level between the LUF group (n = 29) and the ovulation group (n = 152) was increased (9.86 ± 3.83 *vs.* 13.18 ± 3.91 ng/ml, *P* < 0.001, **Figure 2**). The median, minimum, and maximum of progesterone levels in the LUF cycles were all lower than those in the ovulation group.

Pregnancy outcomes of patients in the two groups are shown in **Table 4**. The implantation rate and clinical pregnancy rate were both significantly lower in the LUF group compared with the ovulation group (*P *= 0.004 and *P *= 0.01, respectively). However, early pregnancy loss rate in the LUF group was lower than that in the ovulation group, but the difference was only marginal (*P *= 0.06). Although there was a lower live birth rate in the LUF group (43.50 *vs.* 50.00%), no significant difference was found between the two groups (*P *= 0.16). After adjusting for the luteal support protocols, clinical pregnancy rate was still significantly lower in the LUF group (adjusted *P *= 0.01, OR 0.60, 95% CI 0.42–0.88), while the adjusted *P*-value of early pregnancy loss rate was 0.045 (OR 0.26, 95% CI 0.07–0.97). Furthermore, no differences in pregnancy outcomes were found between various luteal phase support protocols in both groups.

**Table 4:** Pregnancy outcomes between the LUF group and ovulation group.

	LUF	Ovulation	*P*-value	Adjusted *P*-value^a^	OR (95% CI)^b^
Cycles	177	354			
Implantation rate, n (%)	110/267 (41.20)	263/506 (51.97)	0.004		
Clinical pregnancy, n (%)	84 (47.46)	208 (58.76)	0.01	0.01	0.60 (0.42, 0.87)
Early pregnancy loss, n (%)	4 (4.76)	25 (12.02)	0.06	0.045	0.26 (0.07, 0.97)
Live birth, n (%)	77 (43.50)	177 (50.00)	0.16	0.19	0.76 (0.51, 1.14)
Newborn birthweight (g)	3,127.66 ± 580.10	3,060.51 ± 547.63	0.38		

a Binary logistic regression was used to calculate the adjusted P-value with OR (95% CI) and controlled for luteal support protocols and the number of embryos transferred.

b Ovulation group was used as reference.

To further explore the clinical significance of LUF, we subdivided the LUF group by the average LH peak level in LUF cycles (30 IU/L). Baseline characteristics between LH <30 IU/L and LH ≥30 IU/L subgroups were comparable, except for more matured oocytes retrieved from COS cycles in the LH ≥30 IU/L subgroup. Pregnancy outcomes are listed in **Table 5**. The implantation rate, clinical pregnancy rate, and live birth rate were all significantly lower in the LH <30 IU/L subgroup, even after adjusting the number of oocytes retrieved from COS cycles. However, no significant difference in pregnancy outcomes in the ovulation subgroups (grouped by the same cutoff value as the LUF cycles) were observed.

**Table 5:** Pregnancy outcomes between the LH <30 and LH ≥30 groups in LUF cycles.

	LH <30 IU/L	LH ≥30 IU/L	*P*-value	Adjusted *P*-value^a^	OR (95% CI)^b^
Cycles^c^	86	61			
Implantation rate, n (%)	44 (34.38)	54 (57.45)	0.001		
Clinical pregnancy, n (%)	37 (43.02)	38 (62.30)	0.02	0.02	0.45 (0.23, 0.87)
Early pregnancy loss, n (%)	3 (8.12)	1 (2.63)	0.34	0.31	3.04 (0.27, 34.15)
Live birth, n (%)	32 (37.21)	36 (59.02)	0.01	0.01	0.40 (0.20, 0.78)
Newborn birthweight (g)^d^	3,308.75 ± 455.25	2,976.94 ± 645.53	0.04		

a Binary logistic regression was used to calculate the adjusted P-value with OR (95% CI) and controlled for the number of mature oocytes.

b LH ≥30 group was used as reference.

c 30 LUF cycles with missed LH peak values.

d 15 cycles in LH <30 IU/L group and 28 cycles in LH ≥30 IU/L group had twin newborns.

## Discussion

The impact of LUF on clinical outcomes is still uncertain owing to a lack of sufficient evidence. In the present study, we demonstrated that LUF negatively affected pregnancy outcomes in NC-FET of blastocysts, particularly when the LH surge level was inadequate.

Ovulation refers to the process by which a mature oocyte is released from a dominant follicle. It is precisely regulated by various sex hormones and factors, including the LH surge, progestogen, prostaglandins, and a series of enzymes for stigma formation (20). Since the first report of LUFS in 1975, many studies have investigated it’s mechanisms and consequences; however, the detailed molecular mechanism of LUF is still unknown. Endocrine disorders and iatrogenic factors such as non-steroidal anti-inflammatory drugs and clomiphene citrate during stimulated ovulation might be the main causes of LUF. Needless to say, pregnancy would not occur if no oocytes were released around the time of natural intercourse or IUI cycles.

For ovulation to take place, a normal LH peak released by the pituitary and a well-developed periovulatory follicle are needed. It has been shown that concentrations of LH receptors in ovarian corpora lutea from LUF cases are significantly lower than those of ovulation cases (21). Other observational studies show that LUFS is associated with an advanced LH surge or low LH peak (18). In the present study, we found that the LH peak was lower in the LUF group, along with a decreased diameter of the dominant follicles at the time of the LH surge, indicating abnormal LH secretion in our LUF cohort, which was consistent with previous studies.

The corpus luteum is responsible for the production of progesterone and estradiol in the luteal phase. Progesterone is essential for endometrial transitions from the proliferative phase to the secretory phase, which are needed for embryo implantation. It is reported that progesterone level in the mid-luteal phase is significantly lower in LUF cycles (7.32 *vs.* 11.17 ng/ml) (22). Our result also demonstrated that progesterone levels on the embryo transfer day were significantly lower in the LUF group under the same LPS protocol, implying deficient luteal function. In the study of Xu et al. (22), unusual implantation windows and impaired endometrial receptivity in LUFS cases were proposed, as the expression intensities of estrogen receptors and progestogen receptors in the endometrium were significantly higher in LUFS patients than those in the ovulation group, while the expression intensities of integrin ανβ3 in the endometrium were significantly lower. Moreover, luteal resistance index, as detected by transvaginal color-pulsed Doppler ultrasound, in LUFS patients was higher than that in women with normal luteal function, which was similar to those with luteal phase defects (23). Therefore, it is possible that LUF is one of the reasons leading to luteal phase defects and adverse pregnancy outcomes.

Consistent with the hypothesis raised above, we did find that the implantation rate and clinical pregnancy rate in the LUF group were significantly lower when compared with the ovulation group. Our results were inconsistent with the results from the only similar study published by Wang et al. (15). The study included 144 cases of LUF cycles and 866 cases of ovulation cycles with Day 3 embryos transferred in Chinese women; they demonstrated that LUF did not affect the clinical outcomes of FET, suggesting that patients of LUF should continue with FET treatment. However, embryo quality was relatively low in their study, for only 31% of patients had good-quality embryos transferred. Furthermore, slow freezing and rapid thawing protocols were applied to all embryos in their study. Considering the lower survival rate of slow freezing, new studies on embryos cryopreserved by vitrification are warranted.

We were surprised that the live birth rate in the LUF group was lower than that in the ovulation group, but without significance. It perhaps resulted from the insufficient sample size, or the lower early pregnancy loss rate in the LUF group. As discussed above, progesterone is of great importance in embryo implantation and early pregnancy maintenance. A recent meta-analysis showed that progesterone administration for luteal phase support following NC-FET was associated with a higher clinical pregnancy rate, while it was not increased by hCG administration (24), indicating that direct progesterone supplementation might be more important in LUF cycles. In terms of the efficacy of different progesterone preparations, results from randomized control trial studies demonstrate that 10 mg of oral dydrogesterone three times daily achieved similar pregnancy outcomes with micronized vaginal progesterone (90 mg Crinone 8%) in modified NC-FET, while a 400 mg vaginal progesterone suppository twice daily achieved a higher ongoing pregnancy rate than 10 mg of oral dydrogesterone twice daily in HRT cycles (25, 26). In the present study, relatively stronger luteal phase support was used in the LUF group, for more cycles with vaginal progesterone such as 90 mg Crinone (8%), or 600 mg Utrogestan daily was used in the LUF group; while a higher percentage of cycles with dydrogesterone 20 mg daily was found in the ovulation group. This may account for the lower early pregnancy loss rate in the LUF group, leading to no significant difference in live birth rate between the two groups. In addition, the difference in early pregnancy loss rate may also be due to a greater number of cycles in the LUF group receiving more than one type of progesterone for LPS. However, because various LPS protocols were used in our cohort, the efficacy of different types and doses of progesterone supplement for patients with LUF needs further investigation. Moreover, due to the retrospective nature of this study, we could not compare the LUF group and the ovulation group under the same LPS protocols, since strengthened luteal phase support was usually given if LUF was confirmed in clinical practice.

As we discussed above, an abnormal LH surge was associated with luteal phase deficiency and abnormal receptor expression of the endometrium in LUF cycles. To further explore the possible impact of LH peak level on pregnancy outcomes, we stratified the LUF group using the average level of 30 IU/L. We found that pregnancy outcomes were negatively affected with an LH peak <30 IU/L. The higher pregnancy rate and live birth rate of the LH ≥30 IU/L subgroup, which were similar to those of the ovulation group, revealed that luteal function and endometrial receptivity might not be affected in LUF cycles with an adequate LH surge.

The strengths of the present study include that our results were based on a relatively large cohort of LUF cycles in blastocyst FET treatment, which has an increasing trend in clinical application. Furthermore, propensity score matching with control group was used to minimize the confounding factors.

However, our study had several limitations. Our data were derived from a single fertility center; therefore, caution should be taken when applying to other centers and regions. Furthermore, the retrospective research might include confounding factors that were difficult to distinguish. Last but not least, we could not determine which type of luteal phase support protocols were the most suitable for LUF patients in FET cycles, as various types of protocols were used in our clinic.

## Conclusion

LUF negatively affected clinical outcomes of blastocyst FET, especially when the LH surge was inadequate. Further studies with larger sample sizes or prospective randomized trials are warranted to confirm these findings; the mechanism of LUF is definitely deserving of further profound studies.

## Data Availability Statement

The original contributions presented in the study are included in the article. Further inquiries can be directed to the corresponding author.

## Ethics Statement

The studies involving human participants were reviewed and approved by the Research Ethics Committee of the First Affiliated Hospital, Sun Yat-sen University. Written informed consent from the patients/participants was not required to participate in this study in accordance with the national legislation and the institutional requirements.

## Author Contributions

SL, LL, and YX designed the present study. SL, LL, and MS collected the raw data. TM and BM checked the data. SL and LL analyzed and interpreted all the data. SL, LL, and YX were major contributors in writing the manuscript. CZ and YX revised the manuscript. All authors have agreed to be accountable for the content of the work. All authors contributed to the article and approved the submitted version.

## Funding

This study was supported by grants from the National Key R&D Program of China (no. 2018YFC1003102), the National Natural Science Foundation of China (nos. 82071716 and 81771588), and the Guangzhou Science and Technology Project (no. 201804020087).

## Conflict of Interest

The authors declare that the research was conducted in the absence of any commercial or financial relationships that could be construed as a potential conflict of interest.

The reviewer [RH] declared a shared affiliation, with no collaboration, with the authors to the handling editor at the time of review.

## Publisher’s Note

All claims expressed in this article are solely those of the authors and do not necessarily represent those of their affiliated organizations, or those of the publisher, the editors and the reviewers. Any product that may be evaluated in this article, or claim that may be made by its manufacturer, is not guaranteed or endorsed by the publisher.
